# Bisphenol A Data in NHANES Suggest Longer than Expected Half-Life, Substantial Nonfood Exposure, or Both

**DOI:** 10.1289/ehp.0800376

**Published:** 2009-01-28

**Authors:** Richard W. Stahlhut, Wade V. Welshons, Shanna H. Swan

**Affiliations:** 1 Environmental Health Sciences Center, University of Rochester Medical Center, Rochester, New York, USA;; 2 Department of Biomedical Sciences, University of Missouri-Columbia, Columbia, Missouri, USA;; 3 Department of Obstetrics and Gynecology, University of Rochester Medical Center, Rochester, New York, USA

**Keywords:** bisphenol A, exposure assessment, NHANES, pharmacokinetics

## Abstract

**Background:**

It is commonly stated in the literature on human exposure to bisphenol A (BPA) that food is the predominant BPA exposure source, and that BPA is rapidly and completely cleared from the body. If this is correct, BPA levels in fasting individuals should decrease with increased fasting time.

**Objectives:**

We set out to investigate the relationship between urine BPA concentration and fasting time in a population-based sample.

**Methods:**

We modeled log BPA urine concentration as a function of fasting time, adjusted for urine creatinine and other confounders, in 1,469 adult participants in the 2003–2004 National Health and Nutrition Examination Survey. We estimated the BPA “population-based half-life” (pop½) for a fasting time of 0–24 hr, < 4.5 hr, 4.5–8.5 hr, and > 8.5 hr.

**Results:**

The overall pop½ for the 0- to 24-hr interval was 43 hr [95% confidence interval (CI), 26–119 hr]. Among those reporting fasting times of 4.5–8.5 hr (*n* = 441), BPA declined significantly with fasting time, with a pop½ of 4.1 hr (95% CI, 2.6–10.6 hr). However, within the fasting time intervals of 0–4.5 hr (*n* = 129) and 8.5–24 hr (*n* = 899), we saw no appreciable decline. Fasting time did not significantly predict highest (> 12 ng/mL) or lowest (below limit of detection) BPA levels.

**Conclusions:**

Overall, BPA levels did not decline rapidly with fasting time in this sample. This suggests substantial nonfood exposure, accumulation in body tissues such as fat, or both. Explaining these findings may require experimental pharmacokinetic studies of chronic BPA exposure, further examination of BPA levels and effects in fat, and a search for important nonfood sources.

Bisphenol A (BPA) is a high-volume industrial chemical that is polymerized to form polycarbonate plastics and epoxy resins, and is a constituent of some polyvinyl chloride (PVC) plastics. These materials are used to line metal food and drink containers, as dental sealants, and for manufacturing polycarbonate drink containers. The ester bonds that enable the polymerization of BPA can be broken, particularly under exposure to heat, releasing free BPA (Kang et al. 2006).

BPA has long been described as a weak estrogen due to its low affinity for nuclear estrogen receptors, but recent studies have shown that BPA is equipotent with estradiol in its ability to initiate rapid nongenomic responses from membrane surface receptors (Quesada et al. 2002; Wozniak et al. 2005). Whether BPA can cause human health effects is a matter of some debate; the potential for harm to infants and the fetus is currently considered more likely than harm to adults [National Toxicology Program (NTP) 2008; vom Saal et al. 2007].

The Centers for Disease Control and Prevention (CDC) estimates that 93% of Americans ≥ 6 years of age have detectable urine levels of BPA (parent BPA and conjugated metabolites), based on 2003–2004 data from the National Health and Nutrition Examination Survey (NHANES) (Calafat et al. 2008b). The median BPA for all ages was 2.7 ng/mL, with the lowest creatinine-corrected levels found in males, Mexican Americans, and subjects with higher income.

Exposure is thought to be almost exclusively from food (NTP 2008). For example, Wilson et al. (2007) examined exposure to BPA and other phenols in 275 young children 1.5–5 years of age. They concluded that 99% of BPA exposure was dietary based on measurements of BPA levels from a variety of sources such as food, air, and house dust. Because their preliminary survey did not find BPA in tap water, it was not subsequently measured in the main study (Wilson N, personal communication). Miyamoto and Kotake (2006) modeled Japanese exposures and concluded that 95% of adult male BPA intake was from food and drink, excluding water.

In human adults, BPA is generally described as rapidly metabolized, with elimination thought to be virtually complete within 24 hr of exposure. This conclusion is based primarily on three human experimental studies of acute exposures, although animal studies have generally led to similar conclusions (Vandenberg et al. 2007).

Human pharmacokinetics after single exposures were first reported by Völkel and colleagues (2002). In this study, they gave six volunteers deuterium-labeled BPA (5 mg) by mouth and followed elimination in both blood and urine for 42 hr. Urine levels, measured every 6 hr, peaked 6 hr after administration (19.1 μmol; 4,360 ng/mL); the authors reported a urinary elimination half-life of 5.4 hr. Teeguarden et al. (2005) later used these data in the construction of a physiologically based pharmacokinetic model.

In their second acute exposure study, Völkel et al. (2005) gave a much smaller dose of labeled BPA (25 μg) to six volunteers, and followed blood and urine levels for 7 hr. Urine BPA peaked at 1–3 hr (42 nmol; 9.6 ng/mL), with a reported urinary elimination half-life of 4 hr.

Tsukioka et al. (2004) gave 100 μg oral deuterium-labeled BPA to one subject and measured urine BPA levels for 26.5 hr. Urine BPA peaked at 30 min (90 ng/mL) and returned to near baseline (2.2 ng/mL) in this subject by 5 hr. They subsequently dosed 25 subjects with 50 μg BPA, and found that, on average, 76% of administered BPA was eliminated in the urine within 5 hr.

If the rapid BPA clearance seen in these acute exposure studies also occurs in the chronic exposures experienced by the general population, and if BPA exposure is almost entirely through non-water dietary intake, then BPA levels in the population should be inversely related to fasting time. Fasting time, in this context, is analogous to the time after dosing in an experimental pharmacokinetic study. At the start of fasting, BPA exposure should essentially stop and BPA elimination should follow rapidly. Therefore, participants with long fasting times before urine collection should have substantially lower BPA urine levels than those with shorter fasting times.

In this exploratory study, we present evidence that these NHANES BPA data are not consistent with the current consensus that BPA exposures are both rapidly cleared and almost entirely related to food intake. Instead, it appears plausible that there is substantial nonfood exposure, accumulation in body compartments with long elimination times, or both.

## Methods

### Study population

We used data from the 2003–2004 NHANES for this analysis. NHANES, conducted by the CDC National Center for Health Statistics (Hyattsville, MD), is a multistage, stratified, clustered sample of the civilian, noninstitutionalized U.S. population. Data are acquired through standardized examinations at mobile examination centers throughout the United States, with 30 counties, or groups of contiguous counties, surveyed during each 2-year period. Certain subgroups are sampled at a higher rate than other demographic groups, including older adults, Mexican Americans, non-Hispanic blacks, and low-income persons. A CDC institutional review board approved the study protocol, and informed written consent was obtained from all subjects. Detailed methods have been published elsewhere (CDC 2008).

In NHANES 2003–2004, a random one-third subsample of participants ≥ 6 years of age (*n* = 2,567) was selected for urinary BPA measurements. We excluded ages < 18 (34%) because mean fasting times were substantially shorter than those of adults, and because adjustment for body mass index (BMI) is complex in children and adolescents. We excluded those ≥ 85 years of age (2%) to reduce pharmacokinetic variability and effects of comorbidity. We also excluded participants who reported fasting times > 24 hr (1%) to reduce regression leverage of these fasting time outliers. This left 1,469 participants with complete data for the measures described below.

### Fasting time

Except for insulin-dependent diabetics, all adults were asked to fast, although failing to fast adequately (refusal or mistake) did not disqualify participants. Appointment slips, given to subjects in advance, indicated that once fasting started, subjects should: *a*) “not eat or drink anything except water” and *b*) have “no coffee, tea, food, dietary supplements, mints, cough drops, gum, snacks, or beverages, and no nicotine for at least 3 hours” (CDC 2004b). Participants who attended the morning session were asked to fast 9.5 hr (overnight). Afternoon and evening session subjects were asked to fast 6 hr (CDC 2004a).

At each mobile examination center, phlebotomists assessed subject fasting compliance. Fasting time was calculated by CDC (in hours and minutes) using the participant’s response at the time of blood and urine collection to the question “When was the last time you ate or drank anything other than plain water?” (CDC 2006).

However, phlebotomists were also instructed to consider consumption of “diet soda, black coffee or tea with saccharine or Equal” as equivalent to plain water, and thus these items were permitted during “fasting.” Subjects were also asked whether, since fasting began, they had consumed coffee/tea with cream/sugar, alcohol, gum, mints, lozenges, cough drops, antacids, laxatives, antidiarrheals, or dietary supplements. Consumption of these items was recorded, but without modifying the overall fasting time (CDC 2004b).

### BPA measurement

One spot urine sample was collected from each participant and analyzed by CDC using HPLC and tandem mass spectroscopy to determine BPA concentration (sum of BPA parent compound plus conjugated metabolites; nanograms per milliliter). Samples below the limit of detection (LOD; 0.4 ng/mL) were coded as LOD divided by the square root of 2 (Calafat et al. 2008b).

Detailed BPA analysis methods have been published, including the quality control system that was used to prevent contamination during collection, handling, and analysis of samples (CDC 2007b).

We log-transformed BPA in regression analyses to normalize its distribution. Differences in urine dilution were corrected using urine creatinine. In regressions, this correction was performed by adding to the model the function of urine creatinine that provided the best model fit (urine creatinine and creatinine square root). For descriptive statistics, creatinine-corrected BPA was calculated as BPA divided by urine creatinine.

### BPA levels by fasting hour

To estimate the hour-by-hour trajectory of BPA in the population, we fit linear regression models in which fasting time was represented by 23 dummy variables, each representing a 1-hr interval from 1 to 24 hr with 0–1 hr as the reference group. To plot these results, we used the regression model to predict BPA by hour for a “standard person” having the median of continuous variables and mode of categorical variables. Because creatinine correction in our regressions was achieved by including urine creatinine as a covariate, the model predicts BPA rather than creatinine-corrected BPA. Hence predicted BPA results are given in nanograms per milliliter rather than micrograms per gram creatinine.

### BPA population-based half-lives

Plots of log concentration versus time are commonly used to assess toxicant elimination in experimental pharmacokinetic studies. Under first-order kinetics, in which elimination rate is proportional to internal concentration, the plot of log concentration vs. time is a straight line unless the toxicant distributes from a central compartment (e.g., blood) to a peripheral compartment with a slower elimination rate (e.g., fat). The elimination half-life is computed as −ln(2) divided by the slope (Shen 2007).

In our study, we adapted this approach to a food-based population exposure by regressing log BPA concentration against fasting time, in which each data point represents an individual participant. As in experimental kinetics, we calculated the half-life as –ln(2)/slope, except that in our population-based approach, the slope was represented by the fasting time coefficient from adjusted linear regressions described below. We computed the 95% confidence interval (CI) of the half-life from the lower and upper bounds of the regression coefficient CI in similar fashion (Bryan et al. 1990).

Because we are using pharmacokinetic concepts in a nonstandard epidemiologic manner, we wish to carefully distinguish between experimentally determined kinetics and population-based observations from which kinetics must be inferred. To this end, we use the term “half-life” in its conventional pharmacokinetic sense and the term “population-based half-life” (pop½) for derived kinetics seen in these population data.

In addition to examining the slope over the entire fasting interval (0–24 hr), we also subdivided this time period into three subintervals. We selected cut points that would maximize the slope of the regression line (e.g., result in the shortest pop½) during the period when rapid excretion should be occurring. This was done by fitting regressions to explore a range of intervals from fasting time > 2–5 hr to < 6–10 hr, in half-hour increments (e.g., 2.5–8.5 hr, 3–8.5 hr, 4.5–9 hr, 4.5–9.5 hr). The sharpest slope was found from 4.5–8.5 hr. We therefore defined the fasting hour ranges used in our analyses as 0–4.5 (*n* = 129), 4.5–8.5 (*n* = 441), and 8.5–24 hr (*n* = 899).

### Sensitivity analysis

Fasting time is an important variable in this analysis, but is self-reported. Intentional exaggeration of fasting time could be a problem because subjects were given incentives of $100 for adequate fasting and $70 otherwise. We used serum glucose as a means of identifying subjects at greatest risk of fasting time exaggeration, and repeated our pop½ analyses with these subjects excluded. In subjects with normal glucose tolerance, serum glucose should return to normal fasting level (< 100 mg/dl) within 4–6 hours of eating (Galgani et al. 2006), so we classified subjects as at risk of exaggerated fasting time if they reported fasting ≥ 4.5 hours and had a serum glucose ≥ 100.

Because certain non-water dietary intake was permitted after the start of fasting, we also repeated pop½ analyses after identifying and excluding subjects at risk of important “other-than-water” consumption. We excluded subjects who either *a*) reported consuming, after fasting began, coffee/tea with cream/sugar, alcohol, gum, mints, lozenges, cough drops, antacids, laxatives, antidiarrheals, or dietary supplements, or *b*) were most likely to have consumed “diet soda, black coffee or tea with saccharine or Equal.” Group 1 was specifically recorded in the phlebotomy data and easily excluded. Group 2, however, was not recorded; therefore we used the 24-hr dietary history to identify and exclude subjects who consumed diet soda, coffee, or tea on the previous day (with or without sweeteners).

### Statistical analysis

We performed multiple linear regression analyses, adjusted for fasting time, urine creatinine, sex, age, race, BMI, income, and examination session. Age was computed as age in months divided by 12. Race was categorized as Mexican American, white, black, and other/mixed. Income was represented by the poverty income ratio, the ratio of family income to the poverty line, adjusted for family size (continuous, minimum = 0; top-coded at 5). The examination session attended was coded as morning, afternoon, or evening, with the exact time of visit omitted by CDC to help ensure privacy. We also used logistic regression, with covariates as above, to identify predictors of low BPA levels (BPA < LOD; *n* = 114) as well as the highest BPA levels (BPA > 12 ng/mL; *n* = 115).

To assess the effects of hepatic and renal function, we also repeated these analyses, adding to the model the liver enzyme γ-glutamyl transferase, and glomerular filtration rate, a measure of renal function. We estimated the glomerular filtration rate using the four-variable equation from the Modification of Diet in Renal Disease study (Levey et al. 1999).

In complex surveys such as NHANES, survey design variables are available to enable an analyst to make estimates consistent with those that would result from a random sample of the U.S. population (Lee and Forthofer 2005). Sample weights adjust for oversampling and survey imperfections such as nonresponse bias. Survey cluster variables are used to account for the multilevel sampling strategy, in which, for instance, people from a given neighborhood or county are expected to be more similar to each other than to people from other parts of the country.

In this study, we have omitted survey design variables from our analyses because this approach is most favorable to the standard view of BPA as a rapidly cleared exposure. Survey variables generally have the effect of increasing standard errors and reducing power—in this case, reduced power to detect a slope of BPA concentration versus time that should be decidedly different from zero if the standard view is correct. By choosing not to use the weights and other design adjustments, we retain the statistical power found in a convenience sample analysis, but lose the ability to generalize to the U.S. population as a whole. Survey statisticians have extensively debated under what conditions survey design variables are required in regression analyses (Lee and Forthofer 2005).

R (version 2.7.0) was the principal statistical system used in this study (R Development Core Team 2008). Regression diagnostics were performed with the “car” and “alr3” packages; graphics were created using “ggplot2” (Fox 2008; Weisberg 2008; Wickham 2008). A second analyst replicated key statistics independently using SAS 9.1 (SAS Institute Inc., Cary, NC).

## Results

Table 1 shows participant demographics by fasting time interval for the 1,469 subjects. Of these, 48 subjects (3.3%) reported consuming, after fasting began, coffee/tea with cream/sugar, alcohol, gum, mints, lozenges, cough drops, antacids, laxatives, antidiarrheals, or dietary supplements. The 24-hr dietary history identified 119 subjects (8.1%) who consumed diet soft drinks the previous day, and 836 (56.9%) who consumed coffee or tea. Together, there were 920 (62.6%) potential “other-than-water” dietary consumers during fasting.

### Unadjusted BPA levels vary minimally by fasting time

**Figure 1:**
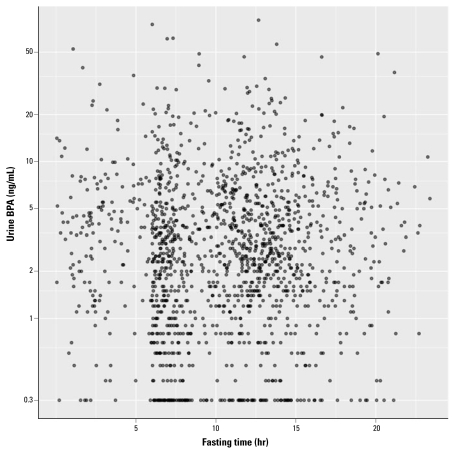
Unadjusted urine BPA level versus fasting time. BPA is not creatinine-corrected; fasting time is self-reported. Points at 0.3 ng/mL BPA are < LOD (see “Methods”).

Table 2 shows unweighted median urinary BPA concentrations, by subgroup, corrected for (i.e., divided by) urine creatinine. Overall, a small decline is seen between 0–4.5 hr and the later fasting time periods (analysis of variance on log-transformed means; *p* = 0.046). Figure 1 is a scatterplot of BPA versus fasting time. Wide scatter is evident without clear trends. The creatinine-corrected scatterplot is similar (not shown).

### BPA kinetics suggested by adjusted models

**Figure 2:**
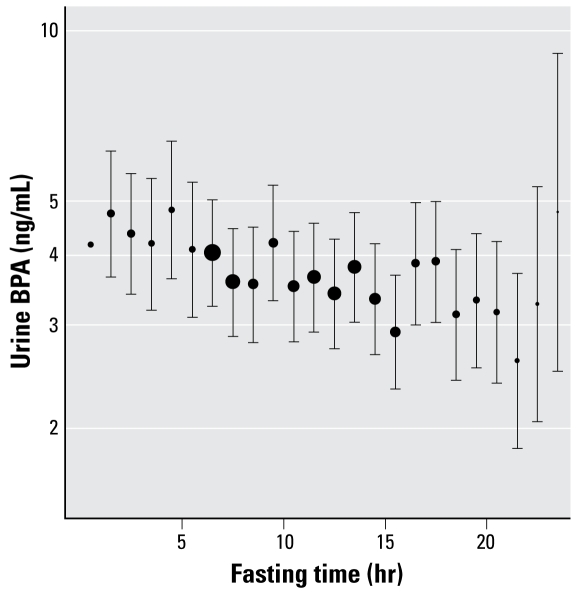
Adjusted urine BPA level versus fasting time. This plot shows BPA levels (± SE) by fasting time hour as determined by linear regression, adjusted for sex, age, race, poverty income ratio, BMI, session, and urine creatinine. Specific BPA levels are computed from the regression based on a “standard” person (see “Results“). SEs appear symmetrical because of the log scale. The size (area) of each point is proportional to the square root of the unweighted *n* (number of observations) in that fasting hour interval.

Figure 2 shows BPA (ln) versus fasting hour, as determined by adjusted linear regression. Individual points were predicted from the regression model using a “standard person” having the median of continuous variables and mode of categorical variables—which for these data was a 44-year-old white female from the morning session with poverty income ratio = 2.1, BMI = 27, and urine creatinine = 122 mg/dL.

Table 3 shows the results of the pop½ regressions, accompanied by an example calculation. The pop½ over the 0–24 hr interval was 43 hr (95% CI, 26 to 119, *p* = 0.002). When regressions were stratified by fasting time intervals, substantial differences in pop½ were seen. For individuals who fasted 0–4.5 hr, the slope was essentially zero, yielding a pop½ of 418 hr (95% CI, 4.7 to −4.8 hr, *p* = 0.98). A nonsignificant trend was also seen in the interval from 8.5 to 24 hours (pop½ = 38 hr; 95% CI, 17 to −225 hr; *p* = 0.09). However, for those fasting 4.5–8.5 hr there was a sharp decline in BPA concentration over the period (pop½ = 4.1 hr; 95% CI, 2.6 to 10.6 hr; *p* = 0.001). Adding gamma-glutamyl transferase and glomerular filtration rate to these models had minimal, nonsignificant effects (results not shown). As shown in Table 3, results did not change substantially after excluding fasting subjects with higher than expected serum glucose, or subjects at risk of “other-than-water” dietary consumption during fasting.

When predictions for the “standard person” were made from a regression over the interval from 4.5 hr (peak) to 24 hr, the BPA level at 4.5 hr was 4.04 ng/mL and at 24 hr was 3.05 ng/mL—a decline of 25% over 19.5 hr. When predictions were made using the peak and trough values taken from Figure 2, the levels declined 46% over 17 hr—from the peak of 4.59 ng/mL at 4–5 hr (*n* = 21) to a trough of 2.48 ng/mL at 21–22 hr (*n* = 19).

### BPA extremes not predicted by fasting time

“BPA < limit of detection” was not predicted by fasting time [odds ratio (OR) = 1.03; 95% CI, 0.98 to 1.09; *p* = 0.28]. Significant predictors were increasing age (continuous years; OR = 1.02; 95% CI, 1.01 to 1.03; *p* = 0.003), race, and urine creatinine. Whites were less likely to be below the LOD than Mexican Americans (OR = 0.38; 95% CI, 0.21–0.69; *p* = 0.001).

“BPA > 12 ng/mL” was not predicted by fasting time (OR = 0.97; 95% CI, 0.93 to 1.01; *p* = 0.15). Significant predictors were female sex (OR = 1.8; 95% CI, 1.2 to 2.8; *p* = 0.005), income (higher income less likely; OR = 0.86; 95% CI, 0.75 to 0.99; *p* = 0.03), and urine creatinine. When only considering long fasting subjects (8.5–24 hr), and excluding at risk subjects as previously described, 7.5% of participants had a BPA level > 12 ng/mL, with a maximum of 80.1 ng/mL.

## Discussion

In these NHANES data, the population-based half-life is much longer than expected based on published acute exposure studies. Regressions demonstrate a strong BPA decline in the 4.5–8.5 hr interval, possibly representing an initial elimination phase subsequent to oral intake. However, from 8.5 to 24 hr, the slope is essentially flat, so pop½ is very long.

The relationship between fasting time and the highest BPA levels (12–80 ng/mL) was weak, and high levels were seen in some long-fasting subjects. Even when restricted to participants fasting 8.5–24 hr, and excluding at risk subjects as previously described, 7.5% of participants had a BPA level > 12 ng/mL. These results are substantially greater than the national median (2.7 ng/mL) determined by Calafat et al. (2008b), and suggest the possibility of important nonfood exposure.

Nonfood exposures have been described. Yamamoto et al. (2000), for example, found substantial migration of BPA from PVC hoses into room temperature water. BPA migration produced substantial BPA levels within 24 hr (median 329 ng/mL). Because PVC pipe is approved for use in residential water supply lines in many cities, BPA exposure from this source (by ingestion or inhalation) deserves investigation. A sizable fraction of this BPA would be chlorinated, however (Yamamoto and Yasuhara 2002). Chlorinated BPA species were not measured in NHANES 2003–2004 (CDC 2007a) or in the children’s study by Wilson et al. (Wilson N, personal communication).

BPA has also been measured in recycled and carbonless copy paper (Ozaki et al. 2004). Reviews by Kang et al. (2006) and Vandenberg et al. (2007) describe other potential sources, such as dental sealants, dust, air, sewage effluents, and landfill leachates.

Although acute, single-dose human experiments give compelling evidence that most BPA is rapidly excreted, frequent exposures could result in accumulation if BPA distributes to tissues that release BPA slowly. The log of the BPA octanol–water partition coefficient (*K*_ow_) has been estimated at 2.2–3.82 (NTP 2008). Thus BPA is lipophilic and could potentially accumulate in fat or other lipid-rich tissues.

BPA levels in fat have been reported. In chronically exposed rodents, Nunez and colleagues (2001) found BPA levels 8–10 times higher in brown fat than in serum. In women, Fernandez et al. (2007) measured BPA in adipose tissue and found, for all subjects, a mean of 3.2 ng BPA/g fat and 8.2 ng chlorinated BPA/g fat. Olea et al. (2008) found substantially greater levels in children. Their mean levels for all subjects were 10.7 ng BPA/g fat and 19.4 chlorinated BPA ng/g fat (Olea N, personal communication). The BPA concentrations from these human studies are much greater than those recently shown to reduce the production of adiponectin in human adipose tissue explants (Hugo et al. 2008). Low adiponectin has been correlated with insulin resistance and vascular inflammatory states (Hajer et al. 2008), whereas total urine BPA (BPA and conjugated metabolites) has been correlated with cardiovascular disease and diabetes (Lang et al. 2008). Thus BPA accumulation in fat, if common, could have important health implications.

Our analysis has several limitations: exclusion of children and the very elderly, self-reported fasting time, non-use of complex survey variables, and the question of BPA contamination or measurement error.

Because we limited our examination to adults 18–85 years of age, these results do not necessarily apply to children or to those > 85 years of age. Although neonates have some capacity to metabolize BPA (Calafat et al. 2008a), neonates and young infants have immature glucuronidation capacity generally (Alcorn and McNamara 2003), which is the primary means by which humans metabolize BPA. In the elderly, changes in body composition and a decline in renal function may affect volume of distribution and excretion rates (Turnheim 2003).

The accuracy of self-reported fasting time appears acceptable. Results did not change substantially after excluding fasting subjects with higher than expected serum glucose (i.e., risk of fasting time exaggeration), or those at risk of “other-than-water” dietary BPA exposure during fasting. Although subjects who reported adequate fasting were compensated $30 more than those who did not, “adequate” fasting was defined as 9.5 hr for the morning examination session and 6 hr for the afternoon and evening sessions. Because longer fasting times than these provided no additional compensation, we do not believe intentional exaggeration is a satisfactory explanation of our results beyond these fasting time minimums.

Because we did not use complex survey variables in our primary analyses, these results cannot be precisely generalized to the entire U.S. population. However, because we adjusted for the demographic covariates related to oversampling, the complex survey analysis method proposed by Korn and Graubard (1991) would produce regression coefficients (and thus population-based half-lives) very similar to ours. The difference is that their method, like other such survey methods, increases the standard errors and would make it more difficult to detect a BPA decline with slope different from zero. Our smaller standard errors are thus more similar to those found in typical cross-sectional studies, except that NHANES sampling procedures and geographic coverage are superior.

We do not suspect substantive BPA contamination or measurement error, because CDC methods include a substantial quality control component (CDC 2007b). NHANES 2003–2004 procedures did not include the use of field blanks (Calafat A, personal communication), but we believe contamination during collection was unlikely because *a*) 6.5% of samples were below the LOD, which argues against systematic error across the entire sample, and *b*) BPA extremes were associated with sex, race, age, and income, which argues against substantial random error.

Although we used pharmacokinetic concepts to assess BPA elimination in the population, continued nonfood exposure could completely obscure rapid clearance kinetics. Therefore the pop½ values obtained here should not be taken as true elimination half-lives, but as a method of quantifying the fact that BPA is, unexpectedly, still present despite extended fasting by many NHANES participants.

More precisely, the concept of a population-based half-life may be useful because the decline in population levels after termination of an exposure should match, at least grossly, the rate predicted by current exposure and pharmacokinetic assumptions. Population-based half-life could be inflated by measurement error in population data, and its variance by differences in exposure. Nevertheless, a large discordance between the measured population-based rate and the rate predicted by current assumptions may be a clue that the assumptions are either incomplete or incorrect. Although, in our study, we used this approach to examine a food-based exposure, it could also be applicable whenever a significant population exposure can be abruptly terminated, such as when a pesticide is banned.

Risk assessments for BPA have been based in part on evidence that food is the primary, and almost exclusive, exposure source, and that rapid and complete BPA elimination occurs after exposure (European Chemicals Bureau 2003; European Food Safety Authority 2006; NTP 2008). The persistence of population BPA levels despite extended fasting appears to contradict this evidence. Research to resolve this contradiction could include experimental pharmacokinetic studies of chronic BPA exposure, continued search for important nonfood sources, and further investigation of BPA in human adipose tissue and its effects on adipokine production. If such studies confirm that either or both of the base assumptions are flawed, risk assessments for BPA will require reevaluation.

## Figures and Tables

**Table 1:** Demographic frequencies [no. (%)] and medians,^a^ by fasting time interval.

	Fasting time interval			
Characteristic	0–24 hr	0–4.5 hr	4.5–8.5 hr	8.5–24 hr
Male	710 (48)	64 (50)	222 (50)	424 (47)
Female	759 (52)	65 (50)	220 (50)	474 (53)
Mexican American	314 (21)	31 (24)	88 (20)	195 (22)
White	749 (51)	52 (40)	242 (55)	455 (51)
Black	288 (20)	37 (29)	66 (15)	185 (21)
Other/mixed	118 (8)	9 (7)	46 (10)	63 (7)
Age (years)	44.2	41.3	47.9	43.5
Poverty income ratio	2.1	1.5	2.2	2.1
BMI	27.1	26.6	26.8	27.3
Fasting time (hr)	10.9	2.3	6.9	13.1

a These descriptive statistics are unweighted and thus describe this sample, not the U.S. population generally.

**Table 2:** Urinary BPA concentrations (no. of subjects, median, μg/g creatinine).

	By fasting time								By session					
	0–24 hr		0–4.5 hr		4.5–8.5 hr		8.5–24 hr		Morning		Afternoon		Evening	
	No.	Median	No.	Median	No.	Median	No.	Median	No.	Median	No.	Median	No.	Median
All	1,469	2.3	129	2.6	441	2.3	899	2.4	697	2.4	547	2.2	225	2.4

Sex														

Male	710	2.1	64	2.4	222	2.0	424	2.1	325	2.2	265	2.0	120	2.3
Female	759	2.5	65	2.7	219	2.4	475	2.6	372	2.5	282	2.5	105	2.7

Age (years)														

18–40	630	2.4	61	2.8	168	2.3	401	2.5	294	2.5	218	2.3	118	2.4
40–60	402	2.4	34	2.4	122	2.3	246	2.3	182	2.6	153	2.3	67	2.5
60–85	437	2.0	34	2.4	151	1.9	252	2.1	221	2.1	176	1.9	40	2.3

Race														

Mexican American	314	2.0	31	2.3	88	1.8	195	2.0	142	2.0	127	2.0	45	1.9
White	749	2.4	52	2.7	242	2.3	455	2.5	369	2.5	271	2.2	109	2.7
Black	288	2.6	37	2.9	66	2.5	185	2.7	135	2.8	105	2.6	48	2.4

Poverty income ratio														

0–1.99	703	2.5	75	2.5	197	2.5	431	2.5	324	2.5	276	2.3	103	3.0
2–4.99	532	2.3	38	2.7	175	2.1	319	2.3	250	2.4	204	2.1	78	2.4
5	234	2.1	16	2.7	69	1.8	149	2.2	123	2.2	67	2.1	44	1.9

BMI														

16–25	515	2.4	53	2.5	161	2.3	301	2.3	237	2.4	188	2.2	90	2.4
25–30	470	2.3	26	2.9	138	2.0	306	2.3	228	2.5	183	2.0	59	2.3
30–65	484	2.4	50	2.4	142	2.3	292	2.4	232	2.3	176	2.3	76	2.7

Session														

Morning	697	2.4	43	3.0	7	2.0	647	2.4						
Afternoon	547	2.2	58	2.4	285	2.1	204	2.2						
Evening	225	2.4	28	2.4	149	2.4	48	2.4						

These descriptive statistics are unweighted and thus describe this sample, not the U.S. population generally.

**Table 3:** Pop½ regression results, sensitivity analyses, medians (μg/g creatinine), and example calculation.^a^

Fasting time	All	Other-than-water consumption exclusion^b^	High-glucose exclusion^c^	Both exclusions
0–24 hr				

No.	1,469	549	1,148	465
Median	2.3	2.3	2.3	2.3
Fasting time beta^d^ (95% CI)	−0.0162 (−0.0267 to −0.0058)	−0.0166 (−0.0311 to −0.002)	−0.0182 (−0.0292 to −0.0072)	−0.0176 (−0.0327 to −0.0025)
Pop½ (hr)	42.7 (26 to 119.5)	41.8 (22.3 to 341.4)	38 (23.7 to 95.7)	39.4 (21.2 to 280.2)
*p*-Value	0.002	0.026	0.001	0.023

0–4.5 hr				

No.	129	55	NA	NA
Median	2.6	2.7		
Fasting time beta^d^ (95% CI)	−0.0017 (−0.1467 to 0.1434)	−0.0101 (−0.2578 to 0.2375)		
Pop½ (hr)	418.3 (4.7 to −4.8)	68.4 (2.7 to −2.9)		
*p*-Value	0.98	0.94		

4.5–8.5 hr				

No.	441	147	368	129
Median	2.3	2.1	2.3	2.0
Fasting time beta^d^ (95% CI)	−0.1671 (−0.2685 to −0.0657)	−0.0924 (−0.2455 to 0.0607)	−0.1815 (−0.2924 to −0.0706)	−0.0865 (−0.2466 to 0.0737)
Pop½ (hr)	4.1 (2.6 to 10.6)	7.5 (2.8 to −11.4)	3.8 (2.4 to 9.8)	8.0 (2.8 to −9.4)
*p*-Value	0.0013	0.24	0.0014	0.29

8.5–24 hr				

No.	899	347	651	281
Median	2.4	2.3	2.4	2.3
Fasting time beta^d^ (95% CI)	−0.0184 (−0.0398 to 0.0031)	−0.0220 (−0.0523 to 0.0083)	−0.0256 (−0.0493 to −0.002)	−0.0188 (−0.0515 to 0.0138)
Pop½ (hr)	37.7 (17.4 to −225.4)	31.5 (13.2 to −83)	27.0 (14.1 to 352)	36.8 (13.5 to −50.1)
*p*-Value	0.093	0.16	0.034	0.26

NA, not applicable.

a Sample calculation: 0–24 hr fasting interval for all participants. The adjusted regression gave a fasting time beta (“slope”) of −0.0162 (95% CI, −0.0267 to −0.0058). The pop½ = −ln(2)/−0.0162 = 42.7 hrs. Lower bound of the 95% CI, –ln(2)/−0.0267 = 26.0 hr; upper bound of the 95% CI, −ln(2)/−0.0058 = 119.5 hr. As slope goes to zero, pop½ goes to infinity (i.e., BPA level is constant). A positive slope (increasing BPA with time) translates to “doubling time” and a negative pop½. Minor inconsistencies between slopes and population-based half-lives are attributable to rounding.

b Excludes participants who reported consuming coffee/tea with cream/sugar, alcohol, gum, mints, lozenges, cough drops, antacids, laxatives, antidiarrheals, or dietary supplements after “fasting” began (*n* = 48) or those who consumed diet soft drinks, coffee, or tea the previous day, and were therefore at higher risk of consuming these items during “fasting” (*n* = 920). Some subjects are in both groups.

c Excludes participants with glucose ≥ 100 mg/dL if fasting time ≥ 4.5 hr.

d Fasting time beta coefficient (estimate) in the adjusted linear regressions; units are nanograms per milliliter per hour (natural log).

## References

[b1-ehp-117-784] Alcorn J, McNamara PJ (2003). Pharmacokinetics in the newborn. Adv Drug Deliv Rev.

[b2-ehp-117-784] Bryan M, Zimmerman JJ, Berry WJ (1990). The use of half-lives and associated confidence intervals in biological research. Vet Res Commun.

[b3-ehp-117-784] Calafat AM, Weuve J, Ye X, Jia LT, Hu H, Ringer S (2008a). Exposure to bisphenol A and other phenols in neonatal intensive care unit premature infants. Environ Health Perspect.

[b4-ehp-117-784] Calafat AM, Ye X, Wong L-Y, Reidy JA, Needham LL (2008b). Exposure of the U.S. population to bisphenol A and 4-*tertiary*-octylphenol: 2003–2004. Environ Health Perspect.

[b5-ehp-117-784] CDC (Centers for Disease Control and Prevention) (2004a). NHANES 2003–2004 Interviewer Procedure Manual Chapters 8–15.

[b6-ehp-117-784] CDC (Centers for Disease Control and Prevention) (2004b). NHANES 2003–2004 Laboratory Procedures Manual.

[b7-ehp-117-784] CDC (Centers for Disease Control and Prevention) (2006). NHANES 2003–2004 Phlebotomy Fasting Questionnaire.

[b8-ehp-117-784] CDC (Centers for Disease Control and Prevention) (2007a). Chemicals measured in selected participants for NHANES 2003–2004.

[b9-ehp-117-784] CDC (Centers for Disease Control and Prevention) (2007b). NHANES 2003–2004 Lab 24 Environmental Phenols.

[b10-ehp-117-784] CDC (Centers for Disease Control and Prevention) (2008). NHANES: National Health and Nutrition Examination Survey.

[b11-ehp-117-784] European Chemicals Bureau (2003). European Union Risk Assessment Report: 4,4′-Isopropylidenediphenol (bisphenol-A).

[b12-ehp-117-784] European Food Safety Authority (2006). Opinion of the Scientific Panel on food additives, flavourings, processing aids and materials in contact with food (AFC) related to 2,2-bis(4-hy-droxyphenyl)propane. http://www.efsa.europa.eu/EFSA/efsa_locale-1178620753812_1178620772817.htm.

[b13-ehp-117-784] Fernandez MF, Arrebola JP, Taoufiki J, Navalon A, Ballesteros O, Pulgar R (2007). Bisphenol-A and chlorinated derivatives in adipose tissue of women. Reprod Toxicol.

[b14-ehp-117-784] Fox J (2008). car: Companion to Applied Regression.

[b15-ehp-117-784] Galgani J, Aguirre C, Diaz E (2006). Acute effect of meal glyce-mic index and glycemic load on blood glucose and insulin responses in humans. Nutr J.

[b16-ehp-117-784] Hajer GR, van Haeften TW, Visseren FL (2008). Adipose tissue dysfunction in obesity, diabetes, and vascular diseases. Eur Heart J.

[b17-ehp-117-784] Hugo ER, Brandebourg TD, Woo JG, Loftus J, Alexander JW, Ben-Jonathan N (2008). Bisphenol A at environmentally relevant doses inhibits adiponectin release from human adipose tissue explants and adipocytes. Environ Health Perspect.

[b18-ehp-117-784] Kang JH, Kondo F, Katayama Y (2006). Human exposure to bisphenol A. Toxicology.

[b19-ehp-117-784] Korn EL, Graubard BI (1991). Epidemiologic studies utilizing surveys: accounting for the sampling design. Am J Public Health.

[b20-ehp-117-784] Lang IA, Galloway TS, Scarlett A, Henley WE, Depledge M, Wallace RB (2008). Association of urinary bisphenol A concentration with medical disorders and laboratory abnormalities in adults. JAMA.

[b21-ehp-117-784] Lee ES, Forthofer RN (2005). Analyzing Complex Survey Data.

[b22-ehp-117-784] Levey AS, Bosch JP, Lewis JB, Greene T, Rogers N, Roth D (1999). A more accurate method to estimate glomerular filtration rate from serum creatinine: a new prediction equation. Ann Intern Med.

[b23-ehp-117-784] Miyamoto K, Kotake M (2006). Estimation of daily bisphenol A intake of Japanese individuals with emphasis on uncertainty and variability. Environ Sci.

[b24-ehp-117-784] NTP (National Toxicology Program) (2008). The potential human reproductive and developmental effects of bisphenol A.

[b25-ehp-117-784] Nunez AA, Kannan K, Giesy JP, Fang J, Clemens LG (2001). Effects of bisphenol A on energy balance and accumulation in brown adipose tissue in rats. Chemosphere.

[b26-ehp-117-784] Olea N, Arrebola JP, Taoufiki J, Fernández-Valades R, Prada R, Navea N, Kungolos S, Brebbia CA, Zamorano M (2008). Alkylphenols and bisphenol-A and its chlorinated derivatives in adipose tissue of children. Environmental Toxicology II WIT Transactions on Ecology and the Environment.

[b27-ehp-117-784] Ozaki A, Yamaguchi Y, Fujita T, Kuroda K, Endo G (2004). Chemical analysis and genotoxicological safety assessment of paper and paperboard used for food packaging. Food Chem Toxicol.

[b28-ehp-117-784] Quesada I, Fuentes E, Viso-Leon MC, Soria B, Ripoll C, Nadal A (2002). Low doses of the endocrine disruptor bisphenol-A and the native hormone 17beta-estradiol rapidly activate transcription factor CREB. FASEB J.

[b29-ehp-117-784] R Development Core Team (2008). R: A Language and Environment for Statistical Computing.

[b30-ehp-117-784] Shen DD, Klaassen CD (2007). Toxicokinetics. Casarett & Doull’s Toxicology: The Basic Science of Poisons.

[b31-ehp-117-784] Teeguarden JG, Waechter JM, Clewell HJ, Covington TR, Barton HA (2005). Evaluation of oral and intravenous route pharmacokinetics, plasma protein binding, and uterine tissue dose metrics of bisphenol A: a physiologically based pharmacokinetic approach. Toxicol Sci.

[b32-ehp-117-784] Tsukioka T, Terasawa J, Sato S, Hatayama Y, Makino T, Nakazawa H (2004). Development of analytical method for determining trace amounts of BPA in urine samples and estimation of exposure to BPA. J Environ Chem.

[b33-ehp-117-784] Turnheim K (2003). When drug therapy gets old: pharmacokinetics and pharmacodynamics in the elderly. Exp Gerontol.

[b34-ehp-117-784] Vandenberg LN, Hauser R, Marcus M, Olea N, Welshons WV (2007). Human exposure to bisphenol A (BPA). Reprod Toxicol.

[b35-ehp-117-784] Völkel W, Bittner N, Dekant W (2005). Quantitation of bisphenol A and bisphenol A glucuronide in biological samples by high performance liquid chromatography-tandem mass spectrometry. Drug Metab Dispos.

[b36-ehp-117-784] Völkel W, Colnot T, Csanady GA, Filser JG, Dekant W (2002). Metabolism and kinetics of bisphenol A in humans at low doses following oral administration. Chem Res Toxicol.

[b37-ehp-117-784] vom Saal FS, Akingbemi BT, Belcher SM, Birnbaum LS, Crain DA, Eriksen M (2007). Chapel Hill bisphenol A expert panel consensus statement: integration of mechanisms, effects in animals and potential to impact human health at current levels of exposure. Reprod Toxicol.

[b38-ehp-117-784] Weisberg S (2008). alr3: Methods and Data to Accompany Applied Linear Regression.

[b39-ehp-117-784] Wickham H (2008). ggplot2: an Implementation of the Grammar of Graphics.

[b40-ehp-117-784] Wilson NK, Chuang JC, Morgan MK, Lordo RA, Sheldon LS (2007). An observational study of the potential exposures of preschool children to pentachlorophenol, bisphenol-A, and nonylphenol at home and daycare. Environ Res.

[b41-ehp-117-784] Wozniak AL, Bulayeva NN, Watson CS (2005). Xenoestrogens at picomolar to nanomolar concentrations trigger membrane estrogen receptor-α-mediated Ca^2+^ fluxes and prolactin release in GH3/B6 pituitary tumor cells. Environ Health Perspect.

[b42-ehp-117-784] Yamamoto T (2000). Determination of bisphenol A migrated from polyvinyl chloride hoses by GC/MS. Bunseki Kagaku.

[b43-ehp-117-784] Yamamoto T, Yasuhara A (2002). Chlorination of bisphenol A in aqueous media: formation of chlorinated bisphenol A con-geners and degradation to chlorinated phenolic compounds. Chemosphere.

